# Optimal assembly strategies of transcriptome related to ploidies of eukaryotic organisms

**DOI:** 10.1186/s12864-014-1192-7

**Published:** 2015-02-08

**Authors:** Bin He, Shirong Zhao, Yuehong Chen, Qinghua Cao, Changhe Wei, Xiaojie Cheng, Yizheng Zhang

**Affiliations:** Key Laboratory of Bio-resources and Eco-environment, Ministry of Education, Sichuan Key Laboratory of Molecular Biology and Biotechnology, College of Life Sciences, Sichuan University, 610064 Chengdu, China

**Keywords:** Transcriptome assembly, RNA-Seq, Diploid, Polyploidy, Sweet potato, *Oryza meyeriana*, *Trametes gallica*

## Abstract

**Background:**

Several *de novo* transcriptome assemblers have been developed recently to assemble the short reads generated from the next-generation sequencing platforms and different strategies were employed for assembling transcriptomes of various eukaryotes without genome sequences. Though there are some comparisons among these *de novo* assembly tools for assembling transcriptomes of different eukaryotic organisms, there is no report about the relationship between assembly strategies and ploidies of the organisms.

**Results:**

When we *de novo* assembled transcriptomes of sweet potato (hexaploid), *Trametes gallica* (a diploid fungus), *Oryza meyeriana* (a diploid wild rice), five assemblers, including Edena, Oases, Soaptrans, IDBA-tran and Trinity, were used in different strategies (Single-Assembler Single-Parameter, SASP; Single-Assembler Multiple-Parameters, SAMP; Combined *De novo* Transcriptome Assembly, CDTA, that is multiple assembler multiple parameter). It was found that CDTA strategy has the best performance compared with other two strategies for assembling transcriptome of the hexaploid sweet potato, whereas SAMP strategy with assembler Oases is better than other strategies for assembling transcriptomes of diploid fungus and the wild rice transcriptomes.

**Conclusion:**

Based on the results from ours and others, it is suggested that CDTA strategy is better used for transcriptome assembly of polyploidy organisms and SAMP strategy of Oases is outperformed for those diploid organisms without genome sequences.

**Electronic supplementary material:**

The online version of this article (doi:10.1186/s12864-014-1192-7) contains supplementary material, which is available to authorized users.

## Background

Transcriptome sequencing projects for non-model organisms have revolutionized the field of biology and medical research and impressively enlarged the realm of transcriptomic analyses, because they cost less and are more computationally tractable than full genome sequencing projects [1]. For instance, these new technologies have been efficiently employed in the discovery of new genes [2], the development of new tissue specific or cancer biomarkers [3], the isolation of fast-evolving genes [4], the detection of new alternative splice variants [5], allele-specific gene expression [6], SNP discovery in genes, or epigenetic gene regulation [7].

However, these new sequencing technologies also brought tremendous challenges to traditional *de novo* assembly tools designed for Sanger sequencing, as they are incapable of handling the millions to billions of short reads (35–400 bp each) generated by next-generation sequencing platforms [1, 8]. Meanwhile, the size and quality of the assembled transcriptome seriously affect the subsequent studies. Therefore, several novel *de novo* assembly tools and strategies have been developed, such as ABySS [9], SSAKE [10], Edena [11], Oases [12], Soaptrans [13], Soapdenovo [14], IDBA-tran [15], Trinity [16] and combined *de novo* transcriptome assembly strategy (CDTA) [17, 18]. However, most assemblers and strategies have been employed for assembling transcriptomes of the same species or different organisms with the same ploidies. For instance, Garg et al. compared the performance of Oases, Abyss, Soapdenovo and commercially available CLC Genomics workbench in chickpea (*Cicer arietinum* L. genotype ICC4958) [19]. They obtained the conclusion that the assembly of short-read data set obtained by Oases was found better than others. Zhang et al. employed various assemblers and reported the comparison of *de novo* assembly software tools among Swinepox virus (Swinepox), *Escherichia coli* str. K-12 substr (bacterium), *Saccharomyces cerevisiae* (a diploid yeast) and *Caenorhabditis elegans* (a diploid nematode) [20]. Their conclusion indicated that overlap-layout-consensus (OLC) assemblers are well-suited for very short reads and longer reads of small genomes respectively. For large datasets of more than hundred millions of short reads, De Bruijn graph-based assemblers would be more appropriate. Zhang et al. compared five assemblers (MIRA, Newbler, SOAPdenovo, SOAPdenovo-trans [SOAPtrans], Trinity) to determine the optimal transcriptome sequencing approach in *Geranium maderense* and *Pelargonium x hortorum* [21]. They found that Trinity or SOAPtrans generate high-quality *de novo* transcriptomes with broad coverage. Apparently, different researchers made their own conclusions. In addition, the previous reports on comparsion of assemblers demonstrated the influence of the length of reads, the type of reads and sequencing platform, while ignored the relationship between the assembly strategies and the ploidies of organisms investigated [22–24].

Actually, all transcriptome assembly strategies could be summarized as three types. It was well-known that some assemblers are used in default parameter, such as Trinity, or in optimized parameter, such as CLC genomics workbench. This strategy is called Single-Assembler Single-Parameter (SASP) described below in this paper. It has been known that Trinity is the best assembler in SASP strategy. For other assemblers, different parameters could be chosen to assembly transcriptomes. This strategy is called Single-Assembler Multiple-Parameter (SAMP). In this strategy, the data assembled from different parameters were merged and assembled with CAP3 [17]. In the third strategy, called Combined *De novo* Transcriptome Assembly (CDTA), the final transcriptome is obtained from emerging data assembled from different parameters of various assemblers (could be also called Multiple-Assemblers Multiple-Parameters, MSMP). This strategy has been used for the transcriptome assembly of sweet potato (hexaploid) [25]. In our previous study, we found that the CDTA strategy was the best one to assembly the sweet potato transcriptome. However, when the same strategy was applied for the transcriptome assembly of a diploid fungus, *Trametes gallica*, we found that the data were not better than those assembled from SAMP of Oases. Afterwards, very similar results were obtained from the transcriptome assembly of the diploid wild rice, *Oryza meyeriana*. Intuitively, we suspect that the ploidies of species should have a significant impact on choosing *de novo* assemblers and strategies.

Accordingly, in this study, we systematically compared the performance of SASP, SAMP and CDTA strategies in assembling transcriptomes of sweet potato, wild rice and fungus. Based on the results from ours and others, we provided guidelines for the selection of optimal assembly strategy for various eukaryotic organisms with different ploidies and useful information for improving current assemblers and developing new high-performance assemblers.

## Results

### Sequencing of samples

We filtered the sequence data for low-quality reads at high stringency (reads with more than 20% of bases with Phred quality score of ≤10), reads with unknown nucleotides larger than 5% and reads containing primer/adaptor sequence. From *Trametes gallica*, we obtained a total of 13,274,462 paired-end reads with 90 nt in length (66,372,31 from each end), encompassing about 2 GB of sequence data in fastq format. From *Oryza meyeriana*, we obtained a total of 162,133,290 paired-end reads with 90 nt in length (81,066,645 from each end), encompassing about 10 GB of sequence data in fastq format. From *Ipomoea batatas*, we obtained a total of 48,716,884 paired-end reads with 100 nt in length (24,358,442 from each end), encompassing about 4 GB of sequence data in fastq format (Table 1).

**Table 1:** **Summary of data generated for sequencing of samples**

**Species**	**No. of reads**	**No. of nucleotides (nt)**	**Length of reads (nt)**	**Type of reads**
*Trametes gallica*	13,274,462	1,194,704,580	90	Paired-end
*Oryza meyeriana*	162,133,290	14,591,996,100	90	Paired-end
Sweet patato	48,716,884	3,653,766,300	100	Paired-end

### Preliminary evaluation of different assembly strategies in individual species

The *de novo* assembly of transcriptome was carried out with various assemblers and assembly strategies. We made a preliminary assessment for various assemblers at different k-mer lengths and various assembly strategies in each species from N50 value, number of longer than 1000 bp assembled contigs and average contig size.

In *Trametes gallica*, when using SASP strategy at different k-mer lengths, we found the best assembly to be the Oases program for k = 21, as it resulted in the highest N50 length of 1529 bp, the most contigs larger than 1000 bp of 8627 and the largest average contig length of 881 bp. While using SAMP and CDTA strategy, the results presented that SAMP of Oases has the optimum performance with N50 length of 1624, the most contigs larger than 1000 bp of 10024 and the largest average contig length of 908 bp. In addition, CDTA strategy was also well-behaved, just next to SAMP of Oases (Additional file 1: Table S1).

In sweet potato, the IDBA-tran program for k = 45 generates the best results in SASP strategy with the highest N50 length of 1194, the most contigs larger than 1000 bp of 20,609 and the largest average contig length of 967 bp (Additional file 2: Table S2). Both CDTA and SAMP of Oases and IDBA-tran displayed a good performance.

In *Oryza meyeriana*, the Oases program for k = 27 and SAMP of Oases were seem to give the best in SASP and SAMP strategy (Additional file 3: Table S3). CDTA strategy also generated ideal results with N50 length of 1,756, contigs larger than 1000 bp of 76,871 and average contig length of 1,132 bp.

### Performance evaluation by size distribution among various species

To determine the relation of assemble strategies and species, the information of size distribution of assembled contigs was compared among three species, including N50 value, number of longer than 1000 bp assembled contigs and average contig size.

**Figure 1:**
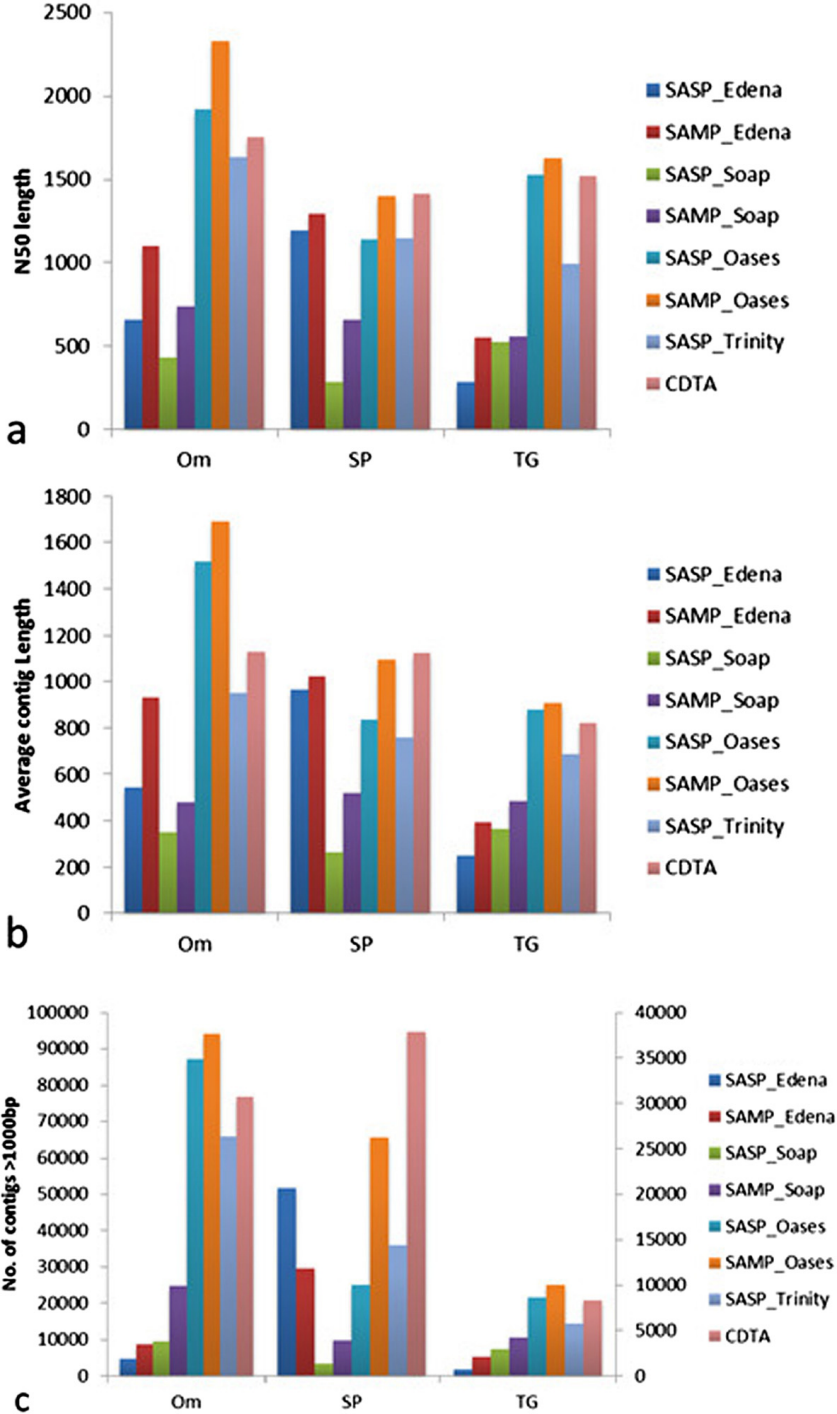
**Comparison of size distribution using various** ***de novo*** **assembly tools and strategies in** ***Trametes gallica*** **, sweet potato and** ***Oryza meyeriana*** **. (a)** Comparison of N50 length. **(b)** Average contig length. **(c)** Number of contigs >1000 bp, data of *Oryza meyeriana* are based on the left Y axes and data of *Trametes gallica* and sweet potato are based on the right Y axes. Note: Edena assembler in sweet potato actually corresponds to IDBA-tran assembler.

The preliminary assessment of these *de novo* assemble strategies showed that evaluation results of SAMP strategy are more advanced than SASP so that we only chose the databases from SAMP strategy and CDTA strategy for further evaluation. The comparison of N50 value showed that SAMP of Oases has a better performance than other strategies in *Trametes gallica* and *Oryza meyeriana*, diploid species, while in sweet potato, hexaploid species, CTDA has better N50 value (Figure 1a). The evaluation results from the average contig size and the number of contigs longer than 1000 bp displayed that the SAMP strategy of Oases was better than the CDTA and other strategies for assembling the transcriptomes of two diploid organisms (Figure 1b) and the CDTA strategy was better than the SAMP for sweet potato transcriptome assembly (Figure 1c).

### Performance evaluation by accuracy and completeness among various species

**Figure 2:**
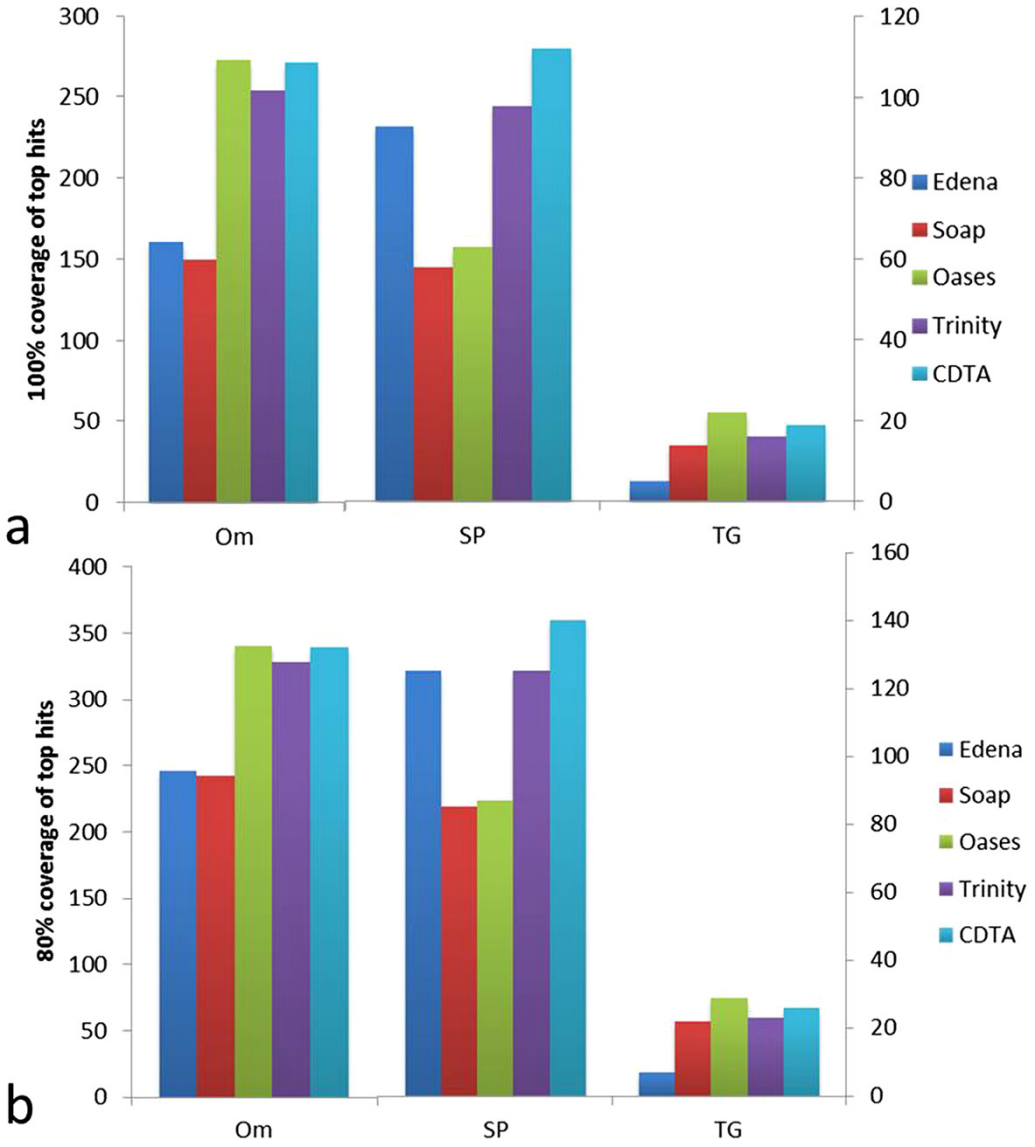
**Comparsion of the length coverage of top database hits using various** ***de novo*** **assembly tools and strategies in** ***Trametes gallica*** **, sweet potato and** ***Oryza meyeriana*** **. (a)** The numbers on 100% of the length coverage of top database hits. **(b)** The numbers on 80% of the length coverage of top database hits. Data of *Oryza meyeriana* are based on the left Y axes and data of *Trametes gallica* and sweet potato are based on the right Y axes. Note: Edena assembler in sweet potato actually corresponds to IDBA-tran assembler.

Another optimality criterion for a novel *de novo* assembled transcriptome is how well it recapitulates previously determined sequences for the target species, and how well it represents sequences from related organisms. The best assembler will return contigs that match previous data well, and will deliver a high coverage of the conserved proteome of related taxa. Through sequence homology search with well-annotated and identified genes in them and phylogenetically related species, we evaluated the accuracy of different *de novo* assemble strategies. Because of the advanced performance of SAMP strategy, we only evaluated the performance of SAMP strategy of multiple k-mer assemblers. The numbers on 100% and 80% of the length coverage of top database hits were counted. The results showed that Oases also has a better performance than other strategies in *Trametes gallica* and *Oryza meyeriana*, although it is not very obvious compared with Trinity and CDTA, while in sweet potato, CTDA strategy performed better than other *de novo* assemblers and strategies (Figure 2). From the accuracy evaluation, CDTA and Oases are more excellent than other *de novo* assemble strategies, especially compared with Soaptrans no matter in *Trametes gallica* and *Oryza meyeriana* or in sweet potato.

**Figure 3:**
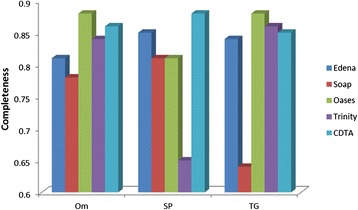
**Comparsion of completeness using various de novo assembly tools and strategies in** ***Trametes gallica*** **, sweet potato and** ***Oryza meyeriana*** **.** Note: Edena assembler in sweet potato actually corresponds to IDBA-tran assembler.

In addition to the statistics of accuracy, completeness was also considered to evaluate the quality of the assemblies. As shown in Figure 3, the value of completeness with the SAMP strategy of Oases is higher than any of other de novo assemblers and strategies in *Trametes gallica* and *Oryza meyeriana*, whereas the contigs provided by the CTDA strategy have the highest value in sweet potato.

### Performance evaluation by long ORF numbers among various species

**Figure 4:**
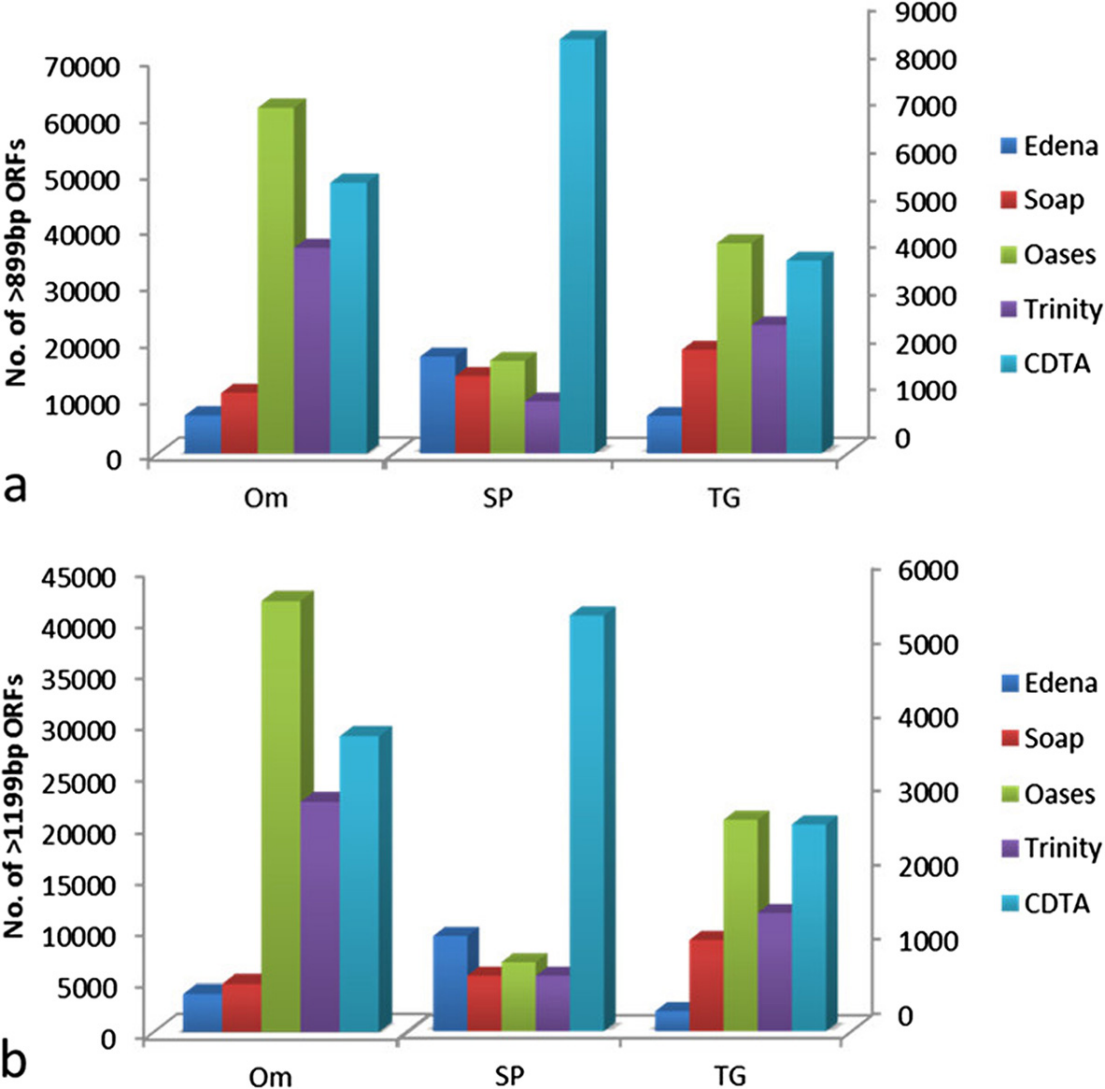
**Comparsion of number of long ORFs using various de novo assembly tools and strategies in** ***Trametes gallica*** **, sweet potato and** ***Oryza meyeriana*** **. (a)** Number of size 900 bp or longer ORFs. **(b)** Number of size 1200 bp or longer ORFs. Data of *Oryza meyeriana* are based on the left Y axes and data of *Trametes gallica* and sweet potato are based on the right Y axes. Note: Edena assembler in sweet potato actually corresponds to IDBA-tran assembler.

Since mRNA was sequenced in RNA-seq and most of mRNA encodes full-length protein, the optimal assembler should produce a large number of long ORFs. To determine the performance of each assembly strategy on long ORF numbers, we count the number of size 900 bp and 1200 bp or longer ORFs. The difference of number of size 900 bp and 1200 bp or longer ORFs is not remarkable and reach a same conclusion. In *Trametes gallica* and *Oryza meyeriana*, SAMP of Oases produced long ORFs at most and exceeded much more than SAMP of Edena and Soaptrans (Figure 4). Meanwhile, CDTA strategy has the best performance in sweet potato and more than twice as SAMP of Oases, even of IDBA-tran, which produced the most long ORFs in SAMP strategy.

## Discussion

With the recent introduction of transcriptome sequencing projects, *de novo* assemblers developed rapidly as well and applied on many species, mainly referring to diploid species and few referring to tetraploid and hexaploid (Table 2). From the previous researches, there was no report particularly interesting in the relationship between ploidies and assembling quality with different *de novo* assemblers and strategies. In this study, different datasets of *Oryza meyeriana*, sweet potato and *Trametes gallica* were generated to address this issue. Our results consistently suggest that the ploidies of species should have a significant impact on the transcriptome quality from different *de novo* assemblers and strategies. During the *de novo* assembly of diploid species, the SAMP strategy of Oases performs the best when comparing with other strategies. Though CDTA strategy gave a similar result with the SAMP strategy of Oases, it spent much more time than the SAMP strategy of Oases (dates not show). While during the *de novo* assembly of hexaploid species, the CDTA strategy shows an obvious advantage than other strategies.

**Table 2:** **Partial application of various assemblers in different ploidies species**

**Species**	**Ploidies**	**Assemblers**	**References**
Chickpea	Diploid	Oases	[19]
Tea plant	Diploid	Soapdenovo	[26]
Carrot	Diploid	CTDA	[17]
Camelina sativa	Diploid	Trinity	[27]
Pasta wheat	Tetraploid	CDTA	[28]
Nicotiana benthamiana	Tetraploid	CDTA	[18]
Common wheat	Hexaploid	Trinity	[29]
Sweetpotato	Hexaploid	Soapdenovo	[30]
Sweetpotato	Hexaploid	CTDA	[25]

To further verify our assembly strategies, we tests our pipelines on other organisms, *Zea mays* (diploid, SRR925467) and *Triticum turgidum* (Tetraploid, SRR863394). 290 and 200 identified mRNA sequences from *Zea mays* and *Triticum turgidum*, respectively, were chosen as reference sequences to evaluate the performance of each assembly strategy. The results showed that our pipelines also applied to other organisms, including diploid and polyploidy species. In *Zea mays*, SAMP of Oases exhibited superiority on N50 value, numbers of contigs longer than 1000 bp and average contig size, especially compared with soap-trans and Trinity (Additional file 4: Table S4). When blasted to the reference genes, Trinity, SAMP of Oases and CDTA strategy all showed a good performance, 144 reference genes were found to be 100% of the length coverage of top database hits (Additional file 5: Figure S1). The results from the numbers of contigs with 80% of the length coverage of top database hits were displayed that SAMP of Oases gave better performance compared with other strategies. In addition, the predicted long ORF numbers and completeness confirmed our speculation as well (Additional file 6: Figure S2). While in *Triticum turgidum*, although the results of CDTA strategy on completeness and numbers of contigs with 100% and 80% of the length coverage of top database hits were similar with that of SAMP of Oases, the performance of CDTA on the length statistics and predicted long ORF numbers were more advanced than other strategies (Additional file 7: Table S5). These results were coincidence with our speculation.

There are many researches displaying very similar results with ours. Transcriptome assembly on Chickpea, diploid (2n = 2× = 16) plant, showed that Oases performs the best comparing with the performance of Abyss, Soapdenovo and commercially available CLC Genomics workbench [19]. Research on optimizing *de novo* assembly of short-read RNA-seq data in *Ricinus communis* showed that SAMP strategy of Oases produced the highest gene coverage among popular assembly packages [31]. In previous transcriptome assembly of sweet potato, CDTA could be a good choice for this hexaploid species, compared with other four assemblers [25]. Furthermore, the research on *Nicotiana benthamiana*, an allo-tetraploid plant, showed that CDTA strategy has a better performance than other strategies [18].

The assembly of the transcriptome of a polyploid species poses additional problems that are not encountered in diploid species. The studies of hexaploid wheat transcriptomes highlight the difficulties of assembling closely related homoeologs in a polyploid species [32]. Schreiber et al. (2012) [33] observed that most homoeologs were collapsed into chimeric contigs when hexaploid wheat transcriptomes were assembled using either Velvet/Oases (60% chimeric sequences) or Trinity (50% chimeric sequences) [33]. Therefore, when mapping reads to the contigs it is important to adjust the number of mismatches to tolerate the average differences generated by genome divergences [32]. In our case, we added additional layers of merging through softwares such as CAP3 with up to 2 nucleotide differences in 40 bp average reads in the first layer. In addition, given assembler-specific optimal parameters, different assemblers can be more efficient at reconstructing different sets of sequences [18]. Therefore, it may be the reason why CDTA strategy has a better performance in the *de novo* assembly of a polyploid species than other strategies but not the SAMP strategy of Oases.

It is undeniable that there exists discrepancy on depths of coverage among three species we chose. However, all of depths of coverage ensures enough genome coverage and control the sequencing error rate because it exceeds the threshold that would affect the transcriptome research [34]. Therefore, in short, we recommended SAMP strategy of Oases to assembly transcriptome of diploid species. Although CDTA strategy has a good performance on three aspects we evaluated above, Oases costs a lot less time than CDTA strategy. While assembling transcriptome of hexaploid and tetraploid species, we recommended CDTA strategy. This conclusion will be vital to those working with transcriptomic data, and will ultimately allow researchers to produce *de novo* assemble high-performance tools for different ploidies of species without genome sequences.

## Conclusion

In this study, diploid species, *Oryza meyeriana* and *Trametes gallica*, and hexaploid species, sweet potato, were sent to transcriptome sequencing to identify the relationship between ploidies and assembling quality with different *de novo* assemblers. We evaluated the performance of each assembly strategy from the size distribution of assembled contigs, the accuracy and the precision and predicted long Open Reading Frame (ORF) numbers. The performance of evaluation have shown that CDTA strategy is better used for transcriptome assembly of polyploidy organisms and SAMP strategy of Oases is outperformed for those diploid organisms. Therefore, this study will be vital to those working with transcriptomic data, and will ultimately allow researchers to produce de novo assemble high-performance tools for different ploidies of species without genome sequences.

## Methods

### Samples and RNA extraction

The fungal strain of Trametes gallica used in this study were activated on potato dextrose agar (PDA) plate and then inoculated cultured statically in 27 kinds of liquid media and cultured statically at 28°C about 10d. Sweet potato [*I. Batatas* (L.) Lam. cv. *Xushu* 18] was grown under normal conditions in Chengdu, Sichuan Province of China [25]. Samples of leaves were collected after planting. *Oryza meyeriana* was planted at natural temperature and light in Yuanjiang, Yunnan Province of China. Samples of roots, stems and leaves were collected. *Trametes gallica* and *Oryza meyeriana* were diploid species, whereas sweet potato was hexaploid species, designated as TG, Om and SP, respectively. Each samples was snap-frozen immediately in nitrogen and stored at −80°C until further processing.

Total RNAs were extracted from each sample by using the Trizol Reagent (Invitrogen, USA), and treated with DNase I (Fermentas, USA) according to the manufacturer’s instructions. RNA concentrations were measured with Qubit fluorometer (Invitrogen, USA).

### Library construction and Illumina sequencing

Beads with oligo(dT) were used to purify poly(A) mRNA from total RNA. Then, the mRNA was fragmented using a RNA fragmentation kit (Ambion). First strand cDNAs were synthesized using Oligo(dT) primer, then second strand cDNAs were synthesized using RNase H and DNA polymerase I. Double stranded cDNAs were random fragmented using Nebulizer, then repaired and added an adenine base to the 3′ end. Then the paired-end cDNA library was prepared with an insert size of 200 bp and submitted to Illumina GA II platform for sequencing at Beijing Genomics Institute (BGI)-Shenzhen, Shenzhen, China (http://www.genomics.cn).

### Read pre-processing

To ensure that the raw data looks good and there are no problems or biases, pair-end raw reads were performed some simple quality control as implemented by fastqc version 0.10.1 [35], including per base sequence quality, per sequence quality scores, per base sequence content, per base GC content and so on. The reads with low scores of less than 20 at 3′ end were filtered out.

### Assembly and strategies

In order to obtain optimal assembling results, three strategies, SASP, SAMP and CTDA, were employed by using four commonly *de novo* assemblers to assemble transcriptomes of above three organisms.

Trinity_release_20131110 (http://trinityrnaseq.github.io/) [16], which used in default parameter, kmer =25, was used in SASP strategy and its Command-line parameters were “--seqType fq --left Reads_1.fq --right Reads_2.fq --CPU 20”.

Three common multiple k-mer *de novo* assemblers, including Edena V3.130110 (www.genomic.ch/edena.php) [11], Oases V0.2.8 (www.ebi.ac.uk/~zerbino/oases/) [12], Soaptrans Release 1.03 (http://sourceforge.net/projects/soapdenovotrans/files/SOAPdenovo-Trans/) [13] and IDBA-tran V1.1.1 (http://www.cs.hku.hk/~alse/idba_tran) [15], were respectively used in SAMP strategy. Edena was first run by using a set of k-mer values and the contig databases obtained from k-mer 40, 45, 50, 55 and 60 were then merged into one contig database with CAP3 [36]. The same strategy as Edena were employed to make assembly by using Soaptrans, Oases and IDBA-tran. Oases was run by using k-mer values of 21, 23, 25, 27 and 29, and Soaptrans was run by the same strategy using k-mer values including 37, 41, 45, 49 and 53, while IDBA-tran was run by using k-mer values of 41, 43, 45, 47 and 49.

**Figure 5:**
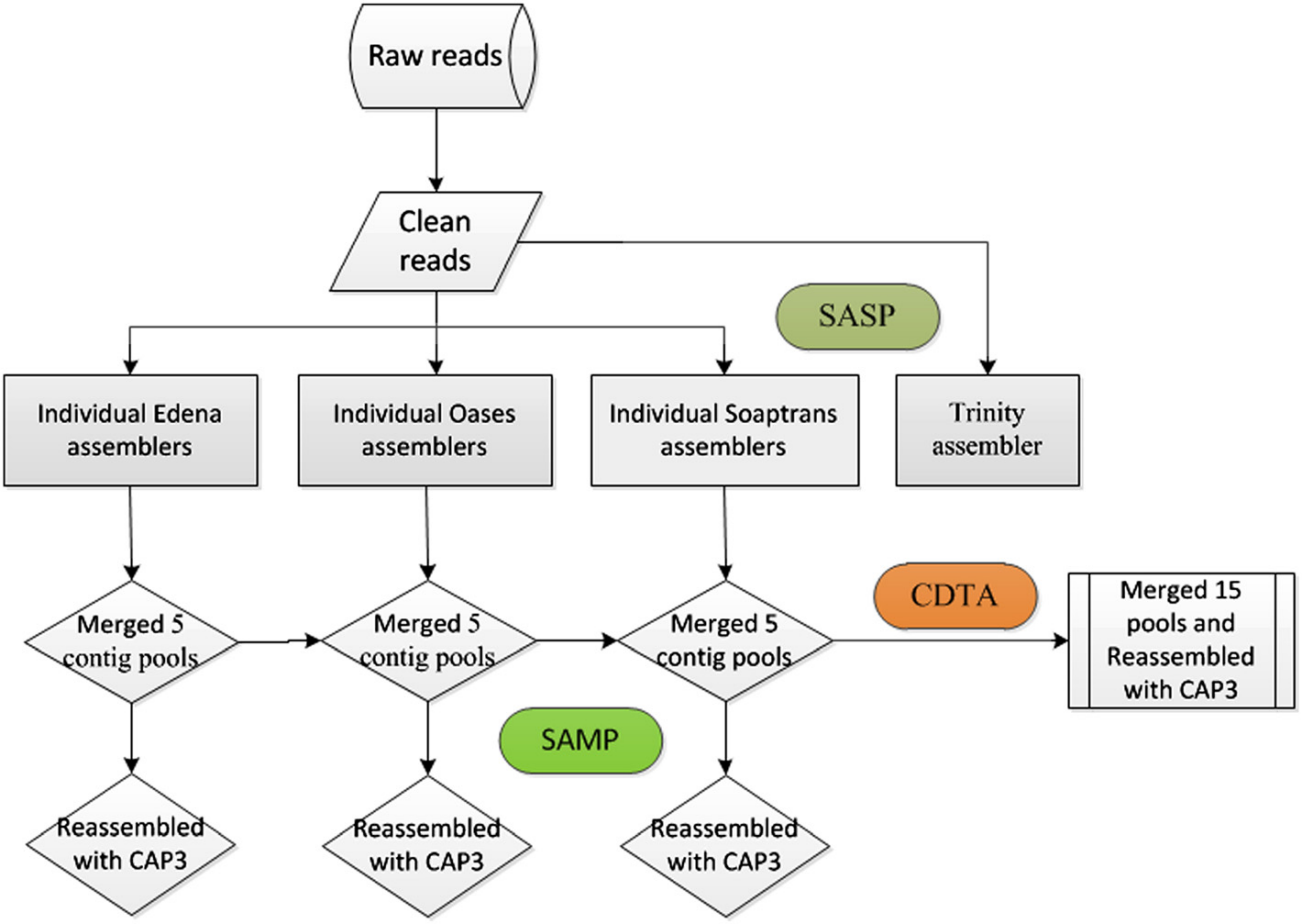
**The detailed pipeline of SASP, SAMP and CDTA strategy.**

In combined *de novo* transcriptome assembly strategy (CTDA), all contig pools from four assemblers described above were merged and reassembled with CAP3. The detailed pipelines of SASP, SAMP and CDTA is shown in Figure 5. All the work of assemblies was run on a 64-bit Linux system (Ubuntu 10.10) with 256G physical memory.

### Performance evaluation

To further evaluate the performance of each assembly strategy, the size distribution of assembled contigs, the accuracy and the precision and predicted long Open Reading Frame (ORF) numbers, were evaluated.

In the first statistics, N50 value (the smallest contig size in which half the assembly is represented) [37, 38], the contig numbers of longer than 1000 bp and the average contig size are always measured as a criterion in evaluating the performance of assemble and generated by common Perl scripts.

To non-model organisms, a sequence homology search, such as by BLASTX, against sequences from a well-annotated, phylogenetically related species is the most practical way to identify the quality of assemble result [39]. The breadth of genetic composition and the transcript contiguity were examined by leveraging a reference data set as accuracy and precision standard [40]. Since the genome of *Trametes gallica*, sweet potato and *Oryza meyeriana* were not available, 159, 312 and 532 identified protein sequences from them and their phylogenetically related species, *Trametes versicolor* laccase and *Oryza sativa* L, were chosen as reference databases. The megablast and the common Perl analysis script were used to analyze the representation.

In addition to the statistics of accuracy, another criterion, completeness, was used to evaluate the quality of the assemblies. Based on the blast results from accuracy evaluation, we considered the average of completeness of each assembly. Completeness, also known as integrity or transcriptome coverage, is the ratio of the sum of all unique aligned segment length to the reference length. We calculated the completeness with Com = TP/(TP + FN) (TP = true positives, FN = false negatives), where Com is completeness, TP is the sum of all aligned segment length (the overlap aligned regions were only calculated once), FN is the sum of all reference segment length that were not aligned.

Since most transcripts assembled from eukaryotic RNA-seq data derived from polyadenylated RNA are expected to code for proteins, the optimal assembly results will produce long and complete ORFs as many as possible. Potential coding regions within reconstructed transcripts were analyzed with the Perl script in the Trinity package. The open reading frames of size 900 bp and 1200 bp or longer were defined as long ORFs in this paper. The percentage of long ORF was compared among different *de novo* assemble tools and strategies.

### Availability of supporting data

The full data sets of *Trametes gallica* have been submitted to NCBI Sequence Read Archive (SRA, http://www.ncbi.nlm.nih.gov/sra/) under Accession SRP050574, Bioproject: PRJNA263488. The full data sets of sweet potato and *Oryza meyeriana* have been submitted to NCBI SRA databases under Accession SRP050169 and SRP050359, Bioproject: PRJNA263487 and PRJNA263485.

## Additional files

Additional file 1: Table S1. Comparison of de novo assembly strategies in *Trametes gallica.*
Additional file 2: Table S2. Comparison of de novo assembly strategies in sweet potato. Additional file 3: Table S3. Comparison of de novo assembly strategies in *Oryza meyeriana.*
Additional file 4: Table S4. Comparison of de novo assembly strategies in *Zea mays.*
Additional file 5: Figure S1. Comparsion of the length coverage of top database hits using various de novo assemblers in *Zea mays* and *Triticum turgidum*. Additional file 6: Figure S2. Comparsion of number of long ORFs numbers and completeness using various de novo assemblers in *Zea mays* and *Triticum turgidum*. The left Zm and Tr indicates the number of size 900 bp or longer ORFs in *Zea mays* and *Triticum turgidum*.The right Zm and Tr indicates the performance of completeness in *Zea mays* and *Triticum turgidum*. Additional file 7: Table S5. Comparison of de novo assembly strategies in *Triticum turgidum.*

## Abbreviations

SASP: Single-Assembler Single-Parameter
SAMP: Single-Assembler Multiple-Parameters
CDTA: Combined De novo Transcriptome Assembly
OLC: Overlap-layout-consensus
ORF: Open Reading Frame
