# Supplementary figures and images for: Optimal assembly strategies of transcriptome related to ploidies of eukaryotic organisms
Source: BMC Genomics. 2015 Feb 8;16(1):65. doi: 10.1186/s12864-014-1192-7 (PMC4343054; doi:10.1186/s12864-014-1192-7)

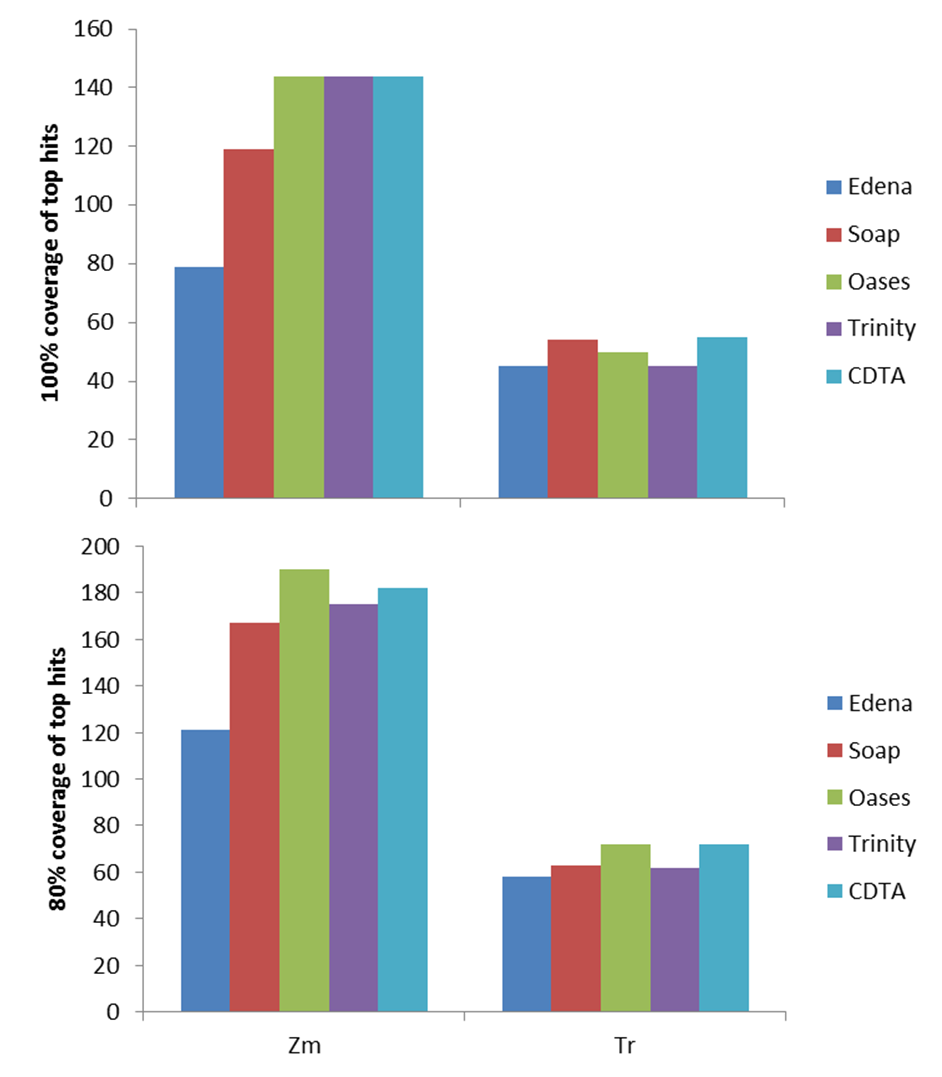

Supplement: Additional file 5: Figure S1. — Comparsion of the length coverage of top database hits using various de novo assemblers in Zea mays and Triticum turgidum. [file 12864_2014_1192_MOESM5_ESM.tiff]

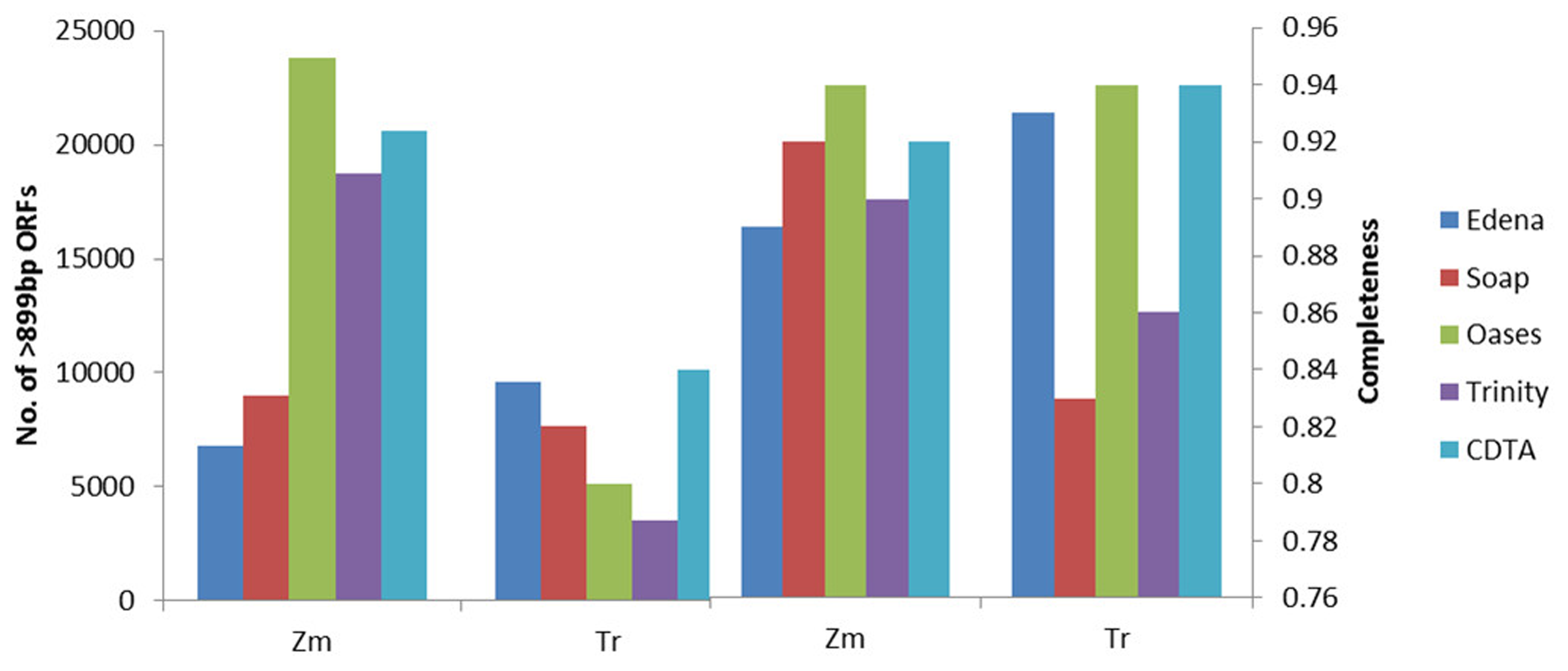

Supplement: Additional file 6: Figure S2. — Comparsion of number of long ORFs numbers and completeness using various de novo assemblers in Zea mays and Triticum turgidum. The left Zm and Tr indicates the number of size 900 bp or longer ORFs in Zea mays and Triticum turgidum.The right Zm and Tr indicates the performance of completeness in Zea mays and Triticum turgidum. [file 12864_2014_1192_MOESM6_ESM.tiff]
